# Supplementary material for: Fusion peptide is superior to co-expressing subunits for arming oncolytic herpes virus with interleukin 12
Source: Commun Med (Lond). 2023 Mar 25;3:40. doi: 10.1038/s43856-023-00270-4 (PMC10039936; doi:10.1038/s43856-023-00270-4)
Supplement: Supplementary file 8 — Supplementary Information [file 43856_2023_270_MOESM8_ESM.pdf]

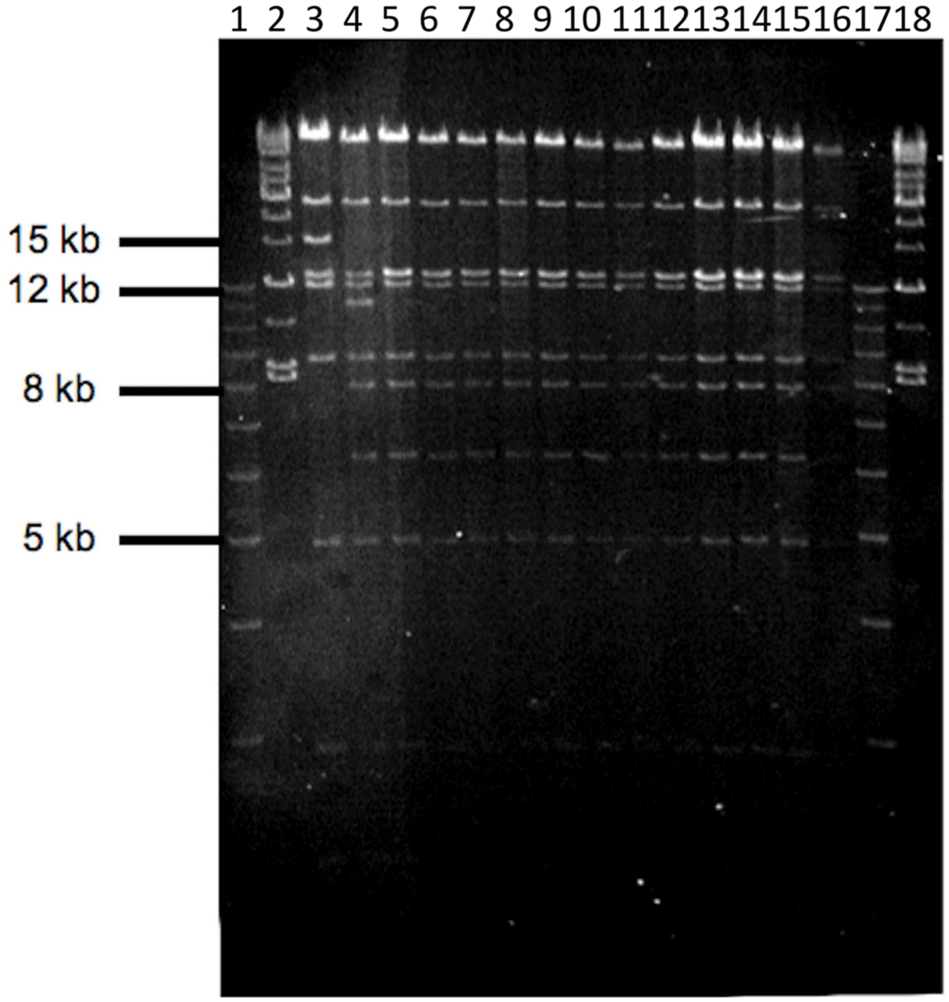

Supplementary Figure 1. Representative gel electrophoresis confirming the structure of T-BAC/Vec9 plasmids obtained after Cre recombination. DNA of T-BAC (lane 3), T-BAC/Vec9-empty (lane 4), No. 1-6 of T-BAC/Vec9-fused-mIL12 (lanes 5-10), or No. 1-6 of T-BAC/Vec9-IRES-mIL12 (lanes 11-16) were digested by *Hin*dIII and separated by electrophoresis on 0.6% (w/v) agarose gels in 1 × Tris-borate-EDTA buffer for 18 h at 2.5 V/cm. All bands are as expected: 15 kb for T-BAC (lane 3), 11 kb, 8 kb, and 6.2 kb for T-BAC/Vec9-empty (lane 4), 8 kb and 6.2 kb for No. 1-6 of T-BAC/Vec9-fused-mIL12 (lanes 5-10), and No. 1-6 of T-BAC/Vec9-IRES-mIL12 (lanes 11-16). Lanes 1, 2, 17, and 18 are kb-markers.

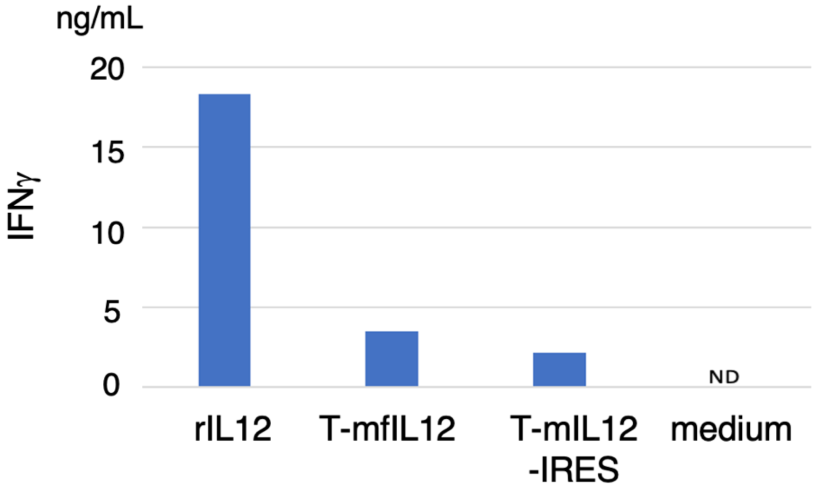

Supplementary Figure 2. Interferon γ release from splenocytes stimulated with virus-encoded IL-12. Supernatants of Vero cells infected with T-mfIL12 or T-mIL12-IRES at multiplicity of infection (MOI)=1 were collected 48 h post-infection. Splenocytes were subjected to the supernatant, recombinant mouse IL-12 or DMEM (medium) for 48 h and interferon-γ (IFNγ) levels were measured (n=2). IL-12 expressed by T-mfIL12 and T-mIL12-IRES both stimulated splenocytes. ND, not detected.

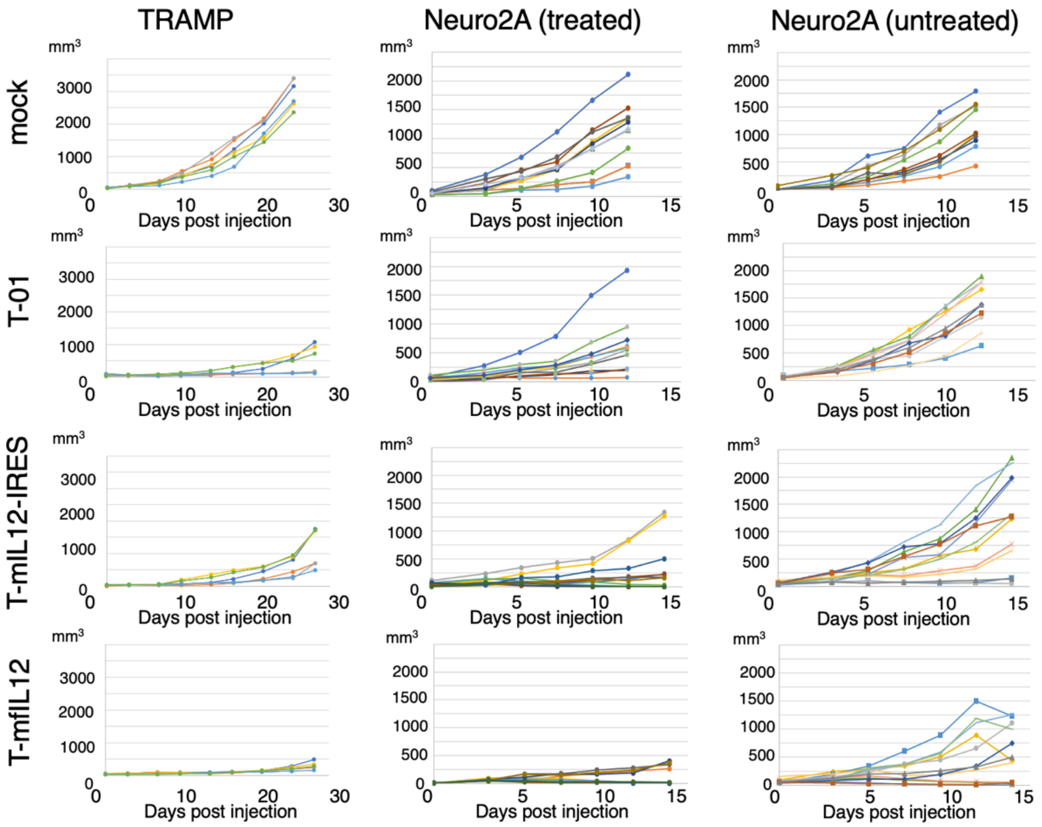

Supplementary Figure 3. Tumor growth curves of individual animals in Figure 4A and 4B.

In the unilateral subcutaneous TRAMP-C2 tumor model, T-01, T-mfIL12, T-mIL12-IRES (5×10^6 pfu) or mock was inoculated intratumorally on days 0 and 3. In the bilateral subcutaneous Neuro2a model, T-01, T-mfIL12, T-mIL12-IRES (5×10^4 pfu) or mock was inoculated into the left tumors only on days 0 and 3.

a

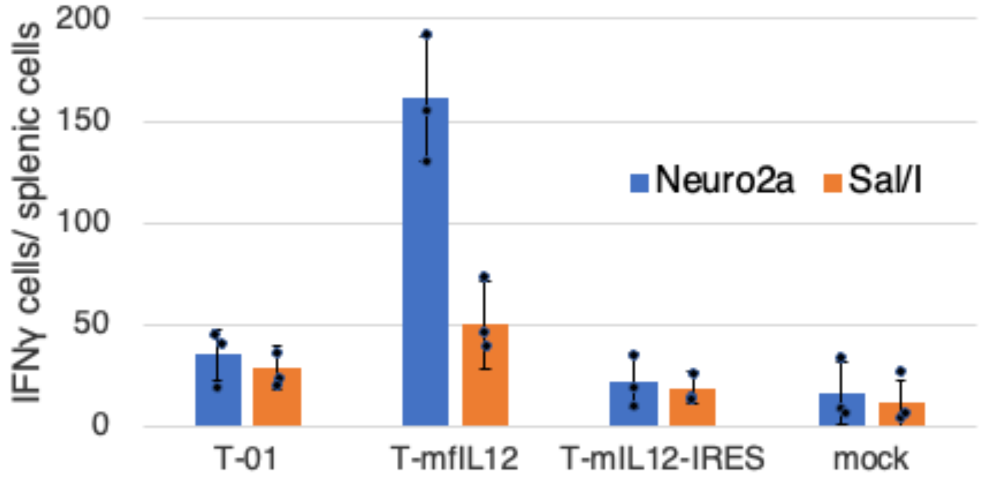

b

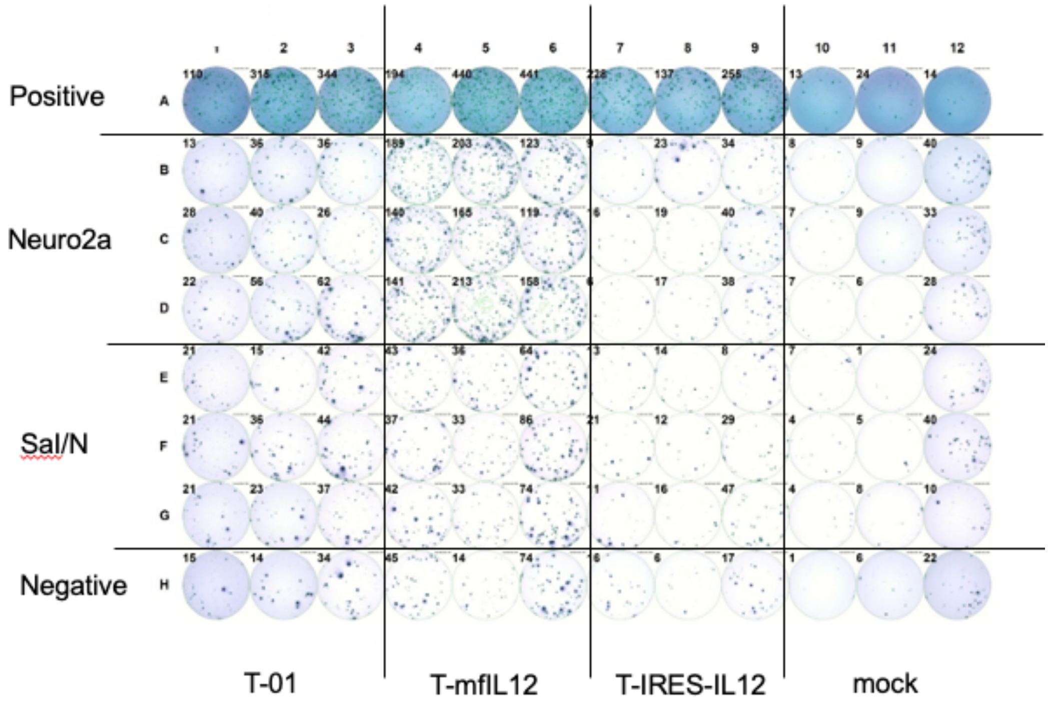

Supplementary Figure 4. ELISpot assay of Interferon γ (IFNγ). Established subcutaneous Neuro2a tumors were inoculated with T-01, T-mfIL12, T-mIL12-IRES (5 x 10^4 pfu) or mock on days 0 and 3, and the spleen was harvested on day 6 (n=3). Splenocytes were stimulated by Neuro2a cells, SaI/N cells, ConA (positive) or no stimulant (negative). **a** Graphs showing the means. Dots represent data from individual mice. **b** A photograph of wells showing the results of ELISpot assay. Bars, SD.

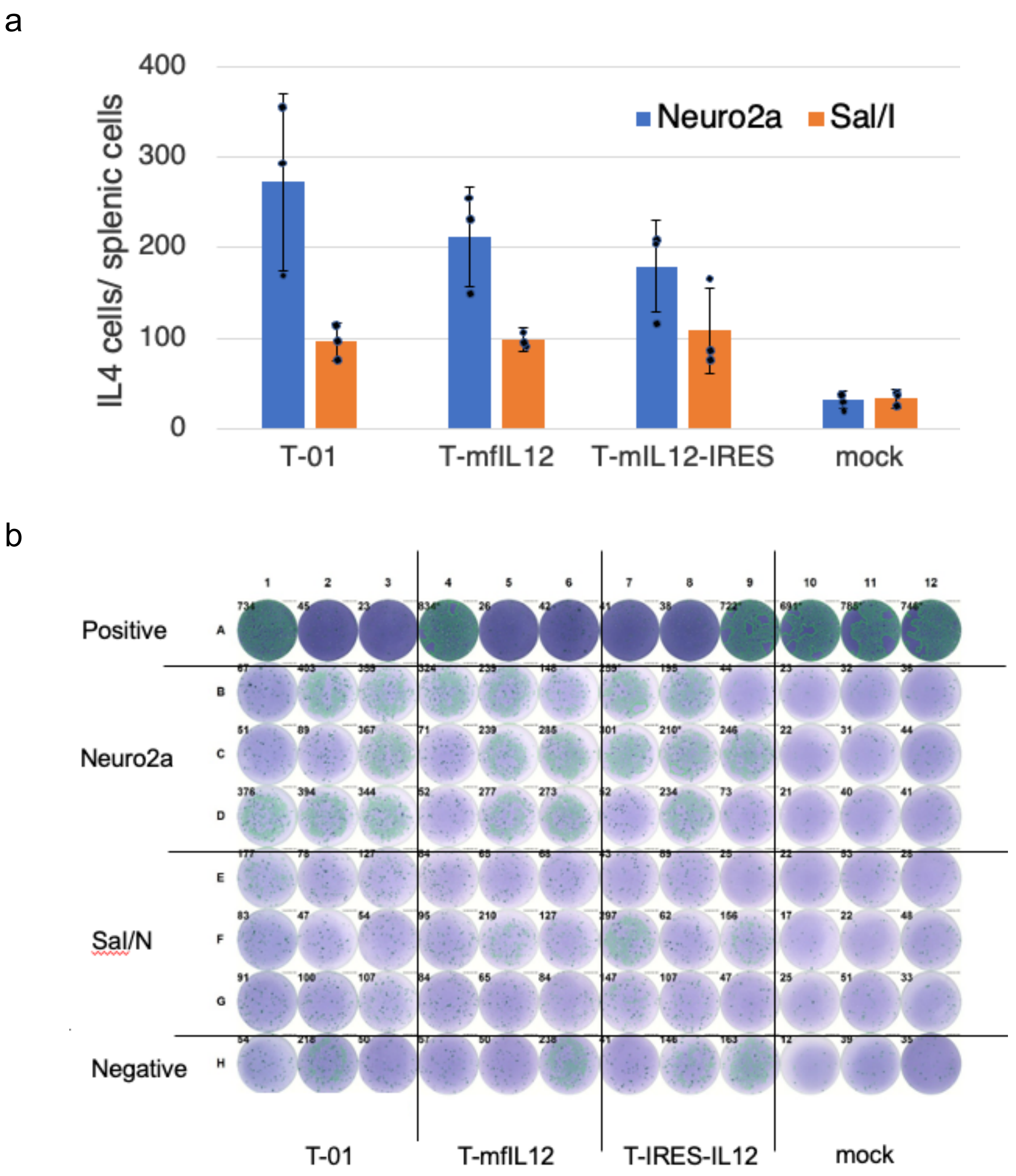

Supplementary Figure 5. ELISpot assay of interleukin-4 (IL-4). Established subcutaneous Neuro2a tumors were inoculated with T-01, T-mfIL12, T-mIL12-IRES (5 x 10^4 pfu) or mock on days 0 and 3, and the spleen was harvested on day 6. Splenocytes were stimulated by Neuro2a cells, SaI/N cells, ConA (positive) or no stimulant (negative). **a** Graphs showing the means. Dots represent data from individual mice. **b** A photograph of wells showing the results of ELISpot assay. Bars, SD.

Supplementary Note 1

T-mfIL12

```
ATGGGTCAATCACGCTACCTCCTCTTTTTGGCCACCCTTGCCCTCCTAAACCACCTCAGTTTGGCCAGG
 M  G  Q  S  R  Y  L  L  F  L  A  T  L  A  L  L  N  H  L  S  L  A  R
GTCATTCCAGTCTCTGGACCTGCCAGGTGTCTTAGCCAGTCCCGAAACCTGCTGAAGACCACAGAT
 V  I  P  V  S  G  P  A  R  C  L  S  Q  S  R  N  L  L  K  T  T  D
GACATGGTGAAGACGGCCAGAGAAAAGCTGAAACATTATTCCTGCACTGCTGAAGACATCGATCAT
 D  M  V  K  T  A  R  E  K  L  K  H  Y  S  C  T  A  E  D  I  D  H
GAAGACATCACACGGGACCAAACCAGCACATTGAAGACCTGTTTACCACTGGAACTACACAAGAAC
 E  D  I  T  R  D  Q  T  S  T  L  K  T  C  L  P  L  E  L  H  K  N
GAGAGTTGCCTGGCTACTAGAGAGACTTCTTCCACAACAAGAGGGAGCTGCCTGCCCCCACAGAAG
 E  S  C  L  A  T  R  E  T  S  S  T  T  R  G  S  C  L  P  P  Q  K
ACGTCTTTGATGATGACCCTGTGCCTTGGTAGCATCTATGAGGACTTGAAGATGTACCAGACAGAG
 T  S  L  M  M  T  L  C  L  G  S  I  Y  E  D  L  K  M  Y  Q  T  E
TTCCAGGCCATCAACGCAGCACTTCAGAATCACAACCATCAGCAGATCATTCTAGACAAGGGCATG
 F  Q  A  I  N  A  A  L  Q  N  H  N  H  Q  Q  I  I  L  D  K  G  M
CTGGTGGCCATCGATGAGCTGATGCAGTCTCTGAATCATAATGGCGAGACTCTGCGCCAGAAACCT
 L  V  A  I  D  E  L  M  Q  S  L  N  H  N  G  E  T  L  R  Q  K  P
CCTGTGGGAGAAGCAGACCCTTACAGAGTGAAAATGAAGCTCTGCATCCTGCTTCACGCCTTCAGC
 P  V  G  E  A  D  P  Y  R  V  K  M  K  L  C  I  L  L  H  A  F  S
ACCCGCGTCGTGACCATCAACAGGGTGATGGGCTATCTGAGCTCCGCCGTTCCTGGAGTAGGGGTA
 T  R  V  V  T  I  N  R  V  M  G  Y  L  S  S  A  V  P  G  V  G  V
CCTGGAGTGGGCGGATCTATGTGGGAGCTGGAGAAAGACGTTTATGTTGTAGAGGTGGACTGGACT
 P  G  V  G  G  S  M  W  E  L  E  K  D  V  Y  V  V  E  V  D  W  T
CCCGATGCCCCTGGAGAAACAGTGAACCTCACCTGTGACACGCCTGAAGAAGATGACATCACCTGG
 P  D  A  P  G  E  T  V  N  L  T  C  D  T  P  E  E  D  D  I  T  W
ACCTCAGACCAGAGGCATGGAGTCATAGGCTCTGGAAAGACCCTGACCATCACTGTCAAAGAGTTT
 T  S  D  Q  R  H  G  V  I  G  S  G  K  T  L  T  I  T  V  K  E  F
CTAGATGCTGGCCAGTACACCTGCCACAAAGGAGGCGAGACTCTGAGCCACTCACATCTGCTGCTC
 L  D  A  G  Q  Y  T  C  H  K  G  G  E  T  L  S  H  S  H  L  L  L
CACAAGAAGGAAAATGGAATTTGGTCCACTGAAATTTTAAAAAATTTCAAAAACAAGACTTTCCTG
 H  K  K  E  N  G  I  W  S  T  E  I  L  K  N  F  K  N  K  T  F  L
AAGTGTGAAGCACCAAATTACTCCGGACGGTTCACGTGCTCATGGCTGGTGCAAAGAAACATGGAC
 K  C  E  A  P  N  Y  S  G  R  F  T  C  S  W  L  V  Q  R  N  M  D
TTGAAGTTCAACATCAAGAGCAGTAGCAGTCCCCCCGACTCTCGGGCAGTGACATGTGGAATGGCG
 L  K  F  N  I  K  S  S  S  S  P  P  D  S  R  A  V  T  C  G  M  A
TCTCTGTCTGCAGAGAAGGTCACACTGGACCAAAGGGACTATGAGAAGTATTCAGTGTCCTGCCAG
 S  L  S  A  E  K  V  T  L  D  Q  R  D  Y  E  K  Y  S  V  S  C  Q
GAGGATGTCACCTGCCCAACTGCCGAGGAGACCCTGCCCATTGAACTGGCGTTGGAAGCACGGCAG
 E  D  V  T  C  P  T  A  E  E  T  L  P  I  E  L  A  L  E  A  R  Q
CAGAATAAATATGAGAACTACAGCACCAGCTTCTTCATCAGGGACATCATCAAACCAGACCCGCCC
 Q  N  K  Y  E  N  Y  S  T  S  F  F  I  R  D  I  I  K  P  D  P  P
AAGAACTTGCAGATGAAGCCTTTGAAGAACTCACAGGTGGAGGTCAGCTGGGAGTACCCTGACTCC
 K  N  L  Q  M  K  P  L  K  N  S  Q  V  E  V  S  W  E  Y  P  D  S
TGGAGCACTCCCCATTCCTACTTCTCCCTCAAGTTCTTTGTTCGAATCCAGCGCAAGAAAGAAAAG
 W  S  T  P  H  S  Y  F  S  L  K  F  F  V  R  I  Q  R  K  K  E  K
ATGAAGGAGACAGAGGAGGGGTGTAACCAGAAAGGTGCGTTCCTCGTAGAGAAGACATCTACCGAA
 M  K  E  T  E  E  G  C  N  Q  K  G  A  F  L  V  E  K  T  S  T  E
GTCCAATGCAAAGGCGGGAATGTCTGCGTGCAAGCTCAGGATCGCTATTACAATTCCTCATGCAGC
 V  Q  C  K  G  G  N  V  C  V  Q  A  Q  D  R  Y  Y  N  S  S  C  S
AAGTGGGCATGTGTTCCCTGCAGGGTCCGATCCTAG
 K  W  A  C  V  P  C  R  V  R  S  *
```

T-mIL12-IRES

ATGTGTCCTCAGAAGCTAACCATCTCCTGGTTTGCCATCGTTTTGCTGGTGTCTCCACTCATGGCCATG
M C P Q K L T I S W F A I V L L V S P L M A M
TGGGAGCTGGAGAAAGACGTTTATGTTGTAGAGGTGGACTGGACTCCCGATGCCCCTGGAGAAACA
W E L E K D V Y V V E V D W T P D A P G E T
GTGAACCTCACCTGTGACACGCCTGAAGAAGATGACATCACCTGGACCTCAGACCAGAGACATGGA
V N L T C D T P E E D D I T W T S D Q R H G
GTCATAGGCTCTGGAAAGACCCTGACCATCACTGTCAAAGAGTTTCTAGATGCTGGCCAGTACACC
V I G S G K T L T I T V K E F L D A G Q Y T
TGCCACAAAGGAGGCGAGACTCTGAGCCACTCACATCTGCTGCTCCACAAGAAGGAAAATGGAATT
C H K G G E T L S H S H L L L H K K E N G I
TGGTCCACTGAAATTTTAAAAAATTTCAAAAACAAGACTTTCCTGAAGTGTGAAGCACCAAATTAC
W S T E I L K N F K N K T F L K C E A P N Y
TCCGGACGGTTCACGTGCTCATGGCTGGTGCAAAGAAACATGGACTTGAAGTTCAACATCAAGAGC
S G R F T C S W L V Q R N M D L K F N I K S
AGTAGCAGTCCCCCCGACTCTCGGGCAGTGACATGTGGAATGGCGTCTCTGTCTGCAGAGAAGGTC
S S S P P D S R A V T C G M A S L S A E K V
ACACTGGACCAAAGGGACTATGAGAAGTATTCAGTGTCCTGCCAGGAGGATGTCACCTGCCCAACT
T L D Q R D Y E K Y S V S C Q E D V T C P T
GCCGAGGAGACCCTGCCCATTGAACTGGCGTTGGAAGCACGGCAGCAGAATAAATATGAGAACTAC
A E E T L P I E L A L E A R Q Q N K Y E N Y
AGCACCAGCTTCTTCATCAGGGACATCATCAAACCAGACCCGCCCAAGAACTTGCAGATGAAGCCT
S T S F F I R D I I K P D P P K N L Q M K P
TTGAAGAACTCACAGGTGGAGGTCAGCTGGGAGTACCCTGACTCCTGGAGCACTCCCCATTCCTAC
L K N S Q V E V S W E Y P D S W S T P H S Y
TTCTCCCTCAAGTTCTTTGTTCGAATCCAGCGCAAGAAAGAAAAGATGAAGGAGACAGAGGAGGGG
F S L K F F V R I Q R K K E K M K E T E E G
TGTAACCAGAAAGGTGCGTTCCTCGTAGAGAAGACATCTACCGAAGTCCAATGCAAAGGCGGGAAT
C N Q K G A F L V E K T S T E V Q C K G G N
GTCTGCGTGCAAGCTCAGGATCGCTATTACAATTCCTCATGCAGCAAGTGGGCATGTGTTCCCTGC
V C V Q A Q D R Y Y N S S C S K W A C V P C
AGGGTCCGATCC**TAG**GATGCAACGGATCCGAATTCCGCCCCCCCCCCCCTAACGTTACNGGCCAAAGC
R V R S *
CCCTTGGAATAAGGCCGGGGGGGGTTTGTCTATATGTTATTTTCCCCCATATTGCCGTCTTTTGGCAT

GGGAGGGCCCGGAAACCTGGCCCTGTCTTCTTGACGAGCATTCCTAGGGGTCTTTCCCCTCTCGCCAA

AGGAATGCAAGGTCTGTTGAATGTCGTGAAGGAAGCAGTTCCTCTGGAAGCTTCTTGAAGACAAACAA

CGTCTGTAGCGACCCTTTGCAGGCAGCGGAACCCCCCACCTGGCGACAGGTGCCTCTGCGGCCAAAA

GCCACGTGTATAAAATACACCTGCAAAGGCGGCACAACCCCAGTGCCACGTTGTGAGTTGGATAGTTG

TGGAAAGAGTCAAATGGCTCTCCTCAAGCGTATTCAACAAGGGGCTGAAGGATGCCCAGAAGGTACCC

CATTGTATGGGATCTGATCTGGGGCCTCGGTGCACATGCTTTACATGTGTTTAGTCGAGGTTAAAAAA

ACGTCTAGGCCCCCCGAACCACGGGGACGTGGTTTTCCTTTGAAAAACACGATGATAATATGCCACAA

CC**ATG**GGTCAATCACGCTACCTCCTCTTTTTGGCCACCCTTGCCCTCCTAAACCACCTCAGTTTGGCC
M G Q S R Y L L F L A T L A L L N H L S L A
AGGGTCATTCCAGTCTCTGGACCTGCCAGGTGTCTTAGCCAGTCCCGAAACCTGCTGAAGACCACA
R V I P V S G P A R C L S Q S R N L L K T T
GATGACATGGTGAAGACGGCCAGAGAAAAGCTGAAACATTATTCCTGCACTGCTGAAGACATCGAT
D D M V K T A R E K L K H Y S C T A E D I D
CATGAAGACATCACACGGGACCAAACCAGCACATTGAAGACCTGTTTACCACTGGAACTACACAAG
H E D I T R D Q T S T L K T C L P L E L H K
AACGAGAGTTGCCTGGCTACTAGAGAGACTTCTTCCACAACAAGAGGGAGCTGCCTGCCCCCACAG
N E S C L A T R E T S S T T R G S C L P P Q

AAGACGTCTTTGATGATGACCCTGTGCCTTGGTAGCATCTATGAGGACTTGAAGATGTACCAGACA
K T S L M M T L C L G S I Y E D L K M Y Q T
GAGTTCCAGGCCATCAACGCAGCACTTCAGAATCACAACCATCAGCAGATCATTCTAGACAAGGGC
E F Q A I N A A L Q N H N H Q Q I I L D K G
ATGCTGGTGGCCATCGATGAGCTGATGCAGTCTCTGAATCATAATGGCGAGACTCTGCGCCAGAAA
M L V A I D E L M Q S L N H N G E T L R Q K
CCTCCTGTGGGAGAAGCAGACCCTTACAGAGTGAAAATGAAGCTCTGCATCCTGCTTCACGCCTTC
P P V G E A D P Y R V K M K L C I L L H A F
AGCACCCGCGTCGTGACCATCAACAGGGTGATGGGCTATCTGAGCTCCGCC**TGA**
S T R V V T I N R V M G Y L S S A *

Supplementary Note. The sequencing data of inserted nucleotides in T-mfIL12 and T-mIL12-IRES. The underlined part of nucleotides in T-mfIL12 corresponds to bovine elastin motifs. In T-mIL12-IRES, the light-green shaded underlined part corresponds to the IRES sequence.
