# Supplementary material for: Fusion peptide is superior to co-expressing subunits for arming oncolytic herpes virus with interleukin 12
Source: Commun Med (Lond). 2023 Mar 25;3:40. doi: 10.1038/s43856-023-00270-4 (PMC10039936; doi:10.1038/s43856-023-00270-4)
Supplement: Supplementary file 10 — Description of Additional Supplementary Files [file 43856_2023_270_MOESM10_ESM.pdf]

Description of Additional Supplementary Files

File Name: Supplementary Data 1

Description: Source data for Figure 2

File Name: Supplementary Data 2

Description: Source data for Supplementary Figure 2

File Name: Supplementary Data 3

Description: Source data for Figure 3

File Name: Supplementary Data 4

Description: Source data for Figure 4

File Name: Supplementary Data 5

Description: Source data for Figure 5, Supplementary Figure 4 and Supplementary Figure 5

File Name: Supplementary Data 6

Description: Source data for Figure 6
